# Perceptions and Attitudes Toward Community Medicine Among Medical Students: A Systematic Review

**DOI:** 10.7759/cureus.52107

**Published:** 2024-01-11

**Authors:** Sunanda Gupta, Aninda Debnath, Shweta Charag, Jugal Kishore

**Affiliations:** 1 Community Medicine, Vardhman Mahavir Medical College and Safdarjung Hospital, New Delhi, IND

**Keywords:** specialty doctor, specialty selection, residency specialty choice, medical specialization, preventive and social medicine, postgraduation, career, medical students, medical education, community medicine

## Abstract

Community medicine is yet to become a popular discipline as a choice for postgraduation and career among medical students in India. Our objective is to find the proportion of students opting for community medicine as a choice for a career. We also aim to find out the perceptions and attitudes of medical students about the subject of community medicine. Our inclusion criteria encompassed studies of any design, written or translated into the English language, and published from their inception up to the last date of our search, which was 15th August 2023. Our comprehensive search covered prominent databases, including PubMed, Scopus, and Embase, as well as an extensive screening of the first 10 pages of Google Scholar and Google. The risk of bias in the studies was evaluated by using the quality assessment tools recommended by the Joanna Briggs Institute critical appraisal tool for prevalence studies. In the initial search, 2069 articles were identified, with 1109 duplicates removed. The remaining 960 articles underwent title and abstract screening, leading to the exclusion of 931 articles. After applying eligibility criteria and reviewing the full text of 29 articles, seven studies were excluded. Ultimately, 22 studies were deemed eligible for inclusion in the systematic review. Among the total of 5106 students, 1032 students expressed a willingness to choose community medicine as their career. The pooled estimate, derived through a random effects model, was 0.21, with a 95% CI of 0.14 to 0.27. Studies conducted in India revealed a willingness of 0.23 (95% CI: 0.13- 0.33), whereas studies conducted outside India reported a lower proportion of 0.17 (0.14-0.24). When considering the year of study, a combined willingness of 0.02 (95% CI: 0.00-0.03) was observed among first and second-year students, contrasting with a higher proportion of 0.18 (95% CI: 0.04-0.32) among third-year students. Fourth-year students and interns demonstrated a willingness of 0.03 (95% CI: 0.00-0.06). The factors for disliking the subject included the perceived absence of clinical engagements, concerns about financial rewards, limited prospects for recognition and fame, etc. By actively engaging in the solution of these challenges, medical educators and policymakers can contribute to the vitalization of community medicine as a coveted and attractive specialty.

## Introduction and background

Community medicine professionals in India find themselves grappling with vulnerability due to the rapid proliferation of schools of public health and departments of family medicine although both are offshoots of preventive and social medicine or community medicine. Among the community medicine fraternity, there lies a strong desire to establish a clinical identity while effectively they are the key persons in integrating a community-focused perspective into health care [1]. This unique quality sets them apart from purely clinical practitioners. Community medicine professionals can establish a beneficial relationship between clinical health care and public health, and hence, the discipline can assert itself as an integral and indispensable aspect of the global healthcare landscape.

It is worth noting that departments of community medicine in India possess well-established infrastructure, encompassing rural and urban field practice areas [2]. This is instrumental in training medical and paramedical students according to national healthcare goals at the community, primary, and secondary levels of health care. Since 2022, in India, the National Medical Commission (NMC) has made it compulsory for medical students (Bachelor of Medicine, Bachelor of Surgery, MBBS) to participate in the Family Adoption Programme (FAP) as a part of their curriculum in community medicine since their first year. The primary goal for students is to bond with these families, comprehend their health conditions and associated determinants, and contribute to enhancing their overall health care and, consequently, that of the broader community [3]. This program is anticipated to play a crucial role in achieving universal health coverage and enhancing early clinical exposure among students [4]. Hence, community medicine is one of the earliest subjects to which medical students are exposed as a part of their course. This also includes patient interaction and role-play of a doctor by the students, which they ultimately aim to become. Despite this, community medicine is yet to become a popular discipline. Our objective is to find the proportion of students opting for community medicine as a choice for a career. We also aim to find out the perceptions and attitudes of medical students about the discipline of community medicine.

## Review

Materials and methods

Research Question and Selection Criteria

The primary objective of our study was to evaluate the inclination of medical students toward choosing community medicine as a career. We included studies conducted among medical students that assessed their willingness to pursue community medicine as a career option. Our inclusion criteria encompassed studies of any design, written or translated into the English language, and published from their inception up to the last date of our search, which was 15th August 2023.

Search Strategy

Two independent authors meticulously conducted a rigorous search to identify studies assessing the willingness of medical students to pursue community medicine as a career. Articles were screened for eligibility based on predefined criteria, and the full-text evaluation was carried out for those meeting the criteria. Any discrepancies during the screening process were resolved through thorough discussions between the authors. In cases where a consensus could not be reached, the third author intervened to ensure resolution. Our comprehensive search covered prominent databases, including PubMed, Scopus, and Embase, as well as an extensive screening of the first 10 pages of Google Scholar. To enhance the search strategy, we utilized keywords such as "Community Medicine," "preventive and social medicine," "preventive medicine," "public health," "medical students," and "medicos." Medical Subject Heading (MeSH) terms were incorporated with an asterisk for further refinement. The deliberate broadness of our search strategy aimed to maximize sensitivity. Additionally, we meticulously examined the reference lists of included studies and other relevant reviews. A secondary search on the first 10 pages of Google Scholar was also conducted to identify any additional eligible studies (Table 1).

**Table 1:** Search strategy MeSH: Medical Subject Heading.

Scopus		
#1	(TITLE-ABS-KEY (community AND medicine) OR TITLE-ABS-KEY (preventive AND social AND medicine))	6,006
#2	TITLE-ABS-KEY (“MBBS Students” OR "MBBS" OR "Undergraduates" OR "Medicos" OR "Medical students" OR "UG")	349,853
#3	#1 AND #2	861
Embase		
#1	('medical student'):ti,ab,kw OR (('MBBS student'):ti,ab,kw) OR (('undergraduate student'):ti,ab,kw)	18,274
#2	('community medicine'):ti,ab,kw OR (('social medicine'):ti,ab,kw) OR (('preventive medicine'):ti,ab,kw)	17,859
#3	#1 AND #2	83
PubMed		
#1	('"community medicine" [Title/Abstract]) OR ("community medicine" [MeSH Terms])) OR ("preventive and social medicine" [MeSH Terms])) OR ("preventive and social medicine" [Title/Abstract])) OR ("preventive medicine" [Title/Abstract])) OR ("preventive medicine" [MeSH Terms])) OR (preventive [Title/Abstract] AND social medicine [Title/Abstract])) OR (preventive and social medicine [MeSH Terms])	46,261
#2	(((((("medical students" [Title/Abstract]) OR ("medical students" [MeSH Terms])) OR (MBBS [MeSH Terms])) OR (MBBS[Title/Abstract])) OR (undergraduate [Title/Abstract])) OR (undergraduate [MeSH Terms])) OR (Undergraduate*[Title/Abstract])) OR (Undergraduate*[MeSH Terms])) OR (medical students [Title/Abstract])) OR (medical students [MeSH Terms])	111,210
#3	#1 AND #2	926
Google Scholar		
#1	allintitle: medical students "community medicine"	130
#2	allintitle: medical students "preventive medicine"	63
#3	allintitle: medical students "preventive and social medicine"	3
#4	#1 OR #2 OR #3	199

Data Extraction and Data Management

We utilized a Microsoft Excel spreadsheet (Microsoft, Redmond, WA) to construct the final data extraction table, encompassing key information such as author details, study location, publication year, study design, and the count of students expressing interest in pursuing community medicine as a career. Emphasis was placed on capturing the proportion of students inclined toward this career choice. Independent data entry into the extraction form was conducted, with meticulous checks for discrepancies. Incomplete data prompted efforts to contact authors for clarification. For citation management and deduplication, Mendeley Desktop V1.19.5 software (Mendeley Ltd., London, UK) was employed. Article screening was independently carried out using the Rayyan application.

Quality Assessment

The risk of bias in the studies was evaluated independently by two authors using the quality assessment tools recommended by the Joanna Briggs Institute critical appraisal tool for prevalence studies [5]. This tool comprises nine questions, strategically designed to assess various aspects of each study. Based on the number of affirmative responses ("yes") to these questions, we categorized the studies into different quality levels. Studies with less than five "'yes" responses were classified as "poor" quality, those with responses between five and six were labeled as "moderate" quality, and studies with more than seven "yes" responses were deemed "high quality." This systematic approach allowed for a comprehensive assessment of the reliability and quality of the included studies.

Data Analysis

The assessment of heterogeneity between studies utilized the chi-square-based Q statistic test and I2 test, with two-sided p-values. Willingness to take community medicine as a career choice was reported along with 95% confidence intervals (CI). The random effects model, based on the DerSimonian and Laird method, was employed for the meta-analysis. In an effort to mitigate bias, a sensitivity analysis was conducted, excluding studies of poor quality. To explore the source of heterogeneity, subgroup analyses were performed based on the study site (India vs. rest of the world), gender, and study year of the participants. Publication bias was evaluated using a funnel plot, and the Egger test was applied to assess small study effects, with a significance level set at p < 0.05. The meta-analysis procedures were executed using STATA® software version 18 (StataCorp LLC, College Station, TX). This comprehensive approach ensures robustness and reliability in the synthesis of study findings.

Results

Search Strategy

**Figure 1:**
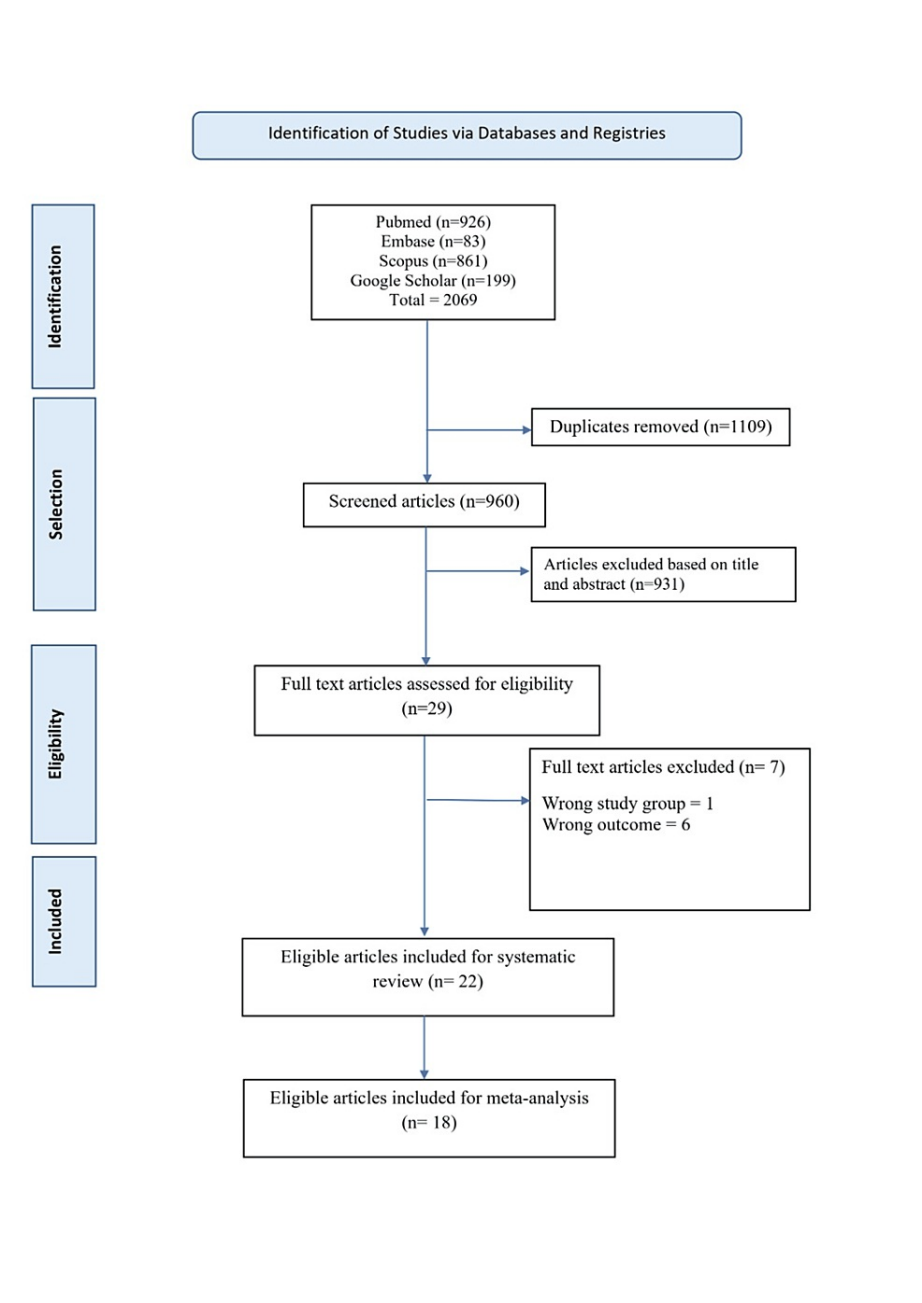
PRISMA chart PRISMA: Preferred Reporting Items for Systematic Reviews and Meta-Analyses.

Apart from the databases PubMed, Embase, and Scopus, articles were searched via Google Scholar. In the initial search, 2069 articles were identified, with 1109 duplicates removed. The remaining 960 articles underwent title and abstract screening, leading to the exclusion of 931 articles. After applying eligibility criteria and reviewing the full text of 29 articles, seven studies were excluded. Ultimately, 22 studies were deemed eligible for inclusion in the systematic review. Notably, four of these studies, which lacked information on the proportion of students willing to pursue community medicine as their career, were excluded from the meta-analysis. The meta-analysis was conducted for the remaining 18 studies, as illustrated in Figure 1.

The studies considered in this analysis spanned from 2009 to 2023 and uniformly adopted a cross-sectional design. Predominantly, the majority of studies (n = 14) originated from India, with three from Nigeria and one each from Canada, Austria, Egypt, Bangladesh, and Pakistan. The sample sizes across the studies exhibited variation, ranging from 57 to 600 individuals. The reported willingness to pursue community medicine as a career varied widely, ranging from 1.5% to 58.6%, as summarized in Table 2. Importantly, all studies included in this analysis underwent quality assessment. Twelve studies were of good quality whereas six studies were of moderate quality (Table 3).

**Table 2:** Characteristics of studies included in the systematic review

S. No.	First author's last name	Year of study	Year of data collection	Name of medical college	State and country	Year of MBBS professional	Total students (N)	Total students willing to take community medicine as a career (n)
1	Trivedi et al. [6]	2023	2022	Government Medical College Bhavnagar	Gujarat, India	Fourth year, internship year	10	Did not report
2	Gupta et al. [7]	2022	2022	Vardhman Mahavir Medical College	New Delhi, India	First year	74	Did not report
3	Prasad et al. [8]	2022	2022	Pt. Jawahar Lal Nehru Memorial Medical College	Chhattisgarh, India	Internship year	28	Did not report
4	Azhar et al. [9]	2022	2021-2022	Combined Military Hospital Lahore Medical College	Lahore, Pakistan	Fourth year, internship year	421	72
5	Muthamilan et al. [10]	2022	2017	Kasturba Medical College, Manipal Academy of Higher Education	Mangalore, India	Second, third, and final years	440	258
6	Ismail et al. [11]	2020	2018-2020	Al-Azhar University	Cairo, Egypt	All year students	600	71
7	Ugwunna et al. [12]	2020	2020	College of Medicine, University of Nigeria	Nigeria	Fourth year	204	90
8	Yadav et al. [13]	2019	2015	D.Y Patil Medical College, Kolhapur	Maharashtra, India	Second and third years	240	53
9	Sengupta et al. [14]	2019	2018-2019	Agartala Government Medical College	Tripura, India	First, second, third, and fourth years	200	8
10	Maiti et al. [15]	2019	2015	Hi-Tech Medical College and Hospital, Bhubaneswar	Odisha, India	First, second, third, fourth, and internship years	398	6
11	Singh et al. [16]	2018	2018	Sarojini Naidu Medical College, Agra	Uttar Pradesh, India	Second, third, and fourth years	316	18
12	Grace et al. [17]	2018	2018	Sree Balaji Medical College and Hospital, Chennai	Tamil Nadu, India	Third year	166	20
13	Murugavel et al. [18]	2017	2015-2016	Tertiary Care Teaching Hospital	Tamil Nadu, India	First, second, third, fourth, and internship years	500	109
14	Sadawarte et al. [19]	2017	2017	Grant Government Medical College	Maharashtra, India	Third year	212	84
15	Egenti et al. [20]	2016	2015-2016	Nnamdi Azikiwe University	Awka, Nigeria	Second and third year	290	41
16	Thakur et al. [21]	2016	2016	Medical College, Indore	Madhya Pradesh, India	All medical students	101	27
17	Singh et al. [22]	2013	2012-2013	Government Medical College	Uttar Pradesh, India	First, second, third, fourth, and internship year	128	20
18	Borsoi et al. [23]	2013	2012	Medical University of Vienna	Vienna, Prague	Fourth year	169	Did not report
19	Nallapu [24]	2012	2010-2011	NRI Medical College, Vijayawada	Andhra Pradesh, India	Third year	244	105
20	Hau et al. [25]	2009	2006	University of Toronto	Toronto, Canada	All medical students	57	18
21	Ossai et al. [26]	2016	2016	Six accredited medical schools	Southeast Nigeria	Final year	457	30
22	Ahmed et al. [27]	2011	2009	Two private medical colleges	Bangladesh	First, third, and fifth years	132	4

**Table 3:** Quality assessment of the studies included in the meta-analysis using the JBI critical appraisal tool for prevalence studies JBI: Joanna Briggs Institute.

Sr. No.	Author	Was the sample frame appropriate to address the target population?	Were study participants sampled in an appropriate way?	Was the sample size adequate?	Were the study subjects and the setting described in detail?	Was the data analysis conducted with sufficient coverage of the identified sample?	Were valid methods used for the identification of the condition?	Was the condition measured in a standard, reliable way for all participants?	Was there an appropriate statistical analysis?	Was the response rate adequate, and if not, was the low response rate managed appropriately?	Total number of "Yes"
1	Azhar et al. [9]	Yes	No	Unclear	Yes	Unclear	Yes	Yes	Yes	Yes	6/9 (Moderate)
2	Muthamilan et al. [10]	Yes	Yes	Unclear	Yes	Unclear	Yes	Yes	Yes	Unclear	6/9 (Moderate)
3	Ismail et al. [11]	Yes	Unclear	Unclear	Yes	Yes	Yes	Yes	Yes	Yes	7/9 (Good)
4	Ugwunna et al. [12]	Unclear	Unclear	Unclear	Yes	Yes	Yes	Yes	Yes	Unclear	5/9 (Moderate)
5	Yadav et al. [13]	Unclear	Unclear	Unclear	Yes	Yes	Yes	Yes	Yes	Unclear	5/9 (Moderate)
6	Sengupta et al. [14]	Yes	Yes	Yes	Yes	Yes	Yes	Yes	Yes	Yes	9/9 (Good)
7	Maiti et al. [15]	Yes	Yes	Yes	Yes	Yes	Yes	Yes	Yes	Yes	9/9 (Good)
8	Singh et al. [16]	Yes	Yes	Yes	Yes	Yes	Yes	Yes	Yes	Yes	9/9 (Good)
9	Grace et al. [17]	Yes	Unclear	Yes	Yes	Yes	Yes	Yes	Yes	Yes	8/9 (Good)
10	Murugavel et al. [18]	Yes	Yes	Yes	Yes	Yes	Yes	Yes	Yes	Yes	9/9 (Good)
11	Sadawarte et al. [19]	Yes	Unclear	Yes	Yes	Yes	Yes	Yes	Yes	Yes	8/9 (Good)
12	Egenti et al. [20]	Yes	Yes	Yes	Yes	Yes	Yes	Yes	Yes	Yes	9/9 (Good)
13	Thakur et al. [21]	Yes	Yes	Yes	Yes	Yes	Yes	Yes	Yes	Yes	9/9 (Good)
14	Singh et al. [22]	Yes	Yes	Yes	Yes	Yes	Yes	Yes	Yes	Yes	9/9 (Good)
15	Nallapu [24]	Yes	Unclear	Yes	Yes	Yes	Yes	Yes	Yes	Yes	8/9 (Good)
16	Hau et al. [25]	Unclear	Unclear	Unclear	Yes	Yes	Yes	Yes	Yes	Yes	6/9 (Moderate)
17	Ossai et al. [26]	Yes	Unclear	Unclear	Yes	Yes	Yes	Yes	Yes	Yes	7/9 (Good)
18	Ahmed et al. [27]	Unclear	Unclear	Unclear	Yes	Yes	Yes	Yes	Yes	Unclear	5/9 (Moderate)

The Cochrane Q-test revealed significant heterogeneity (p < 0.01) with an I2 value of 98.42%. In response to this observed heterogeneity, a random-effects model utilizing the DerSimonian-Laird method was employed.

**Figure 2:**
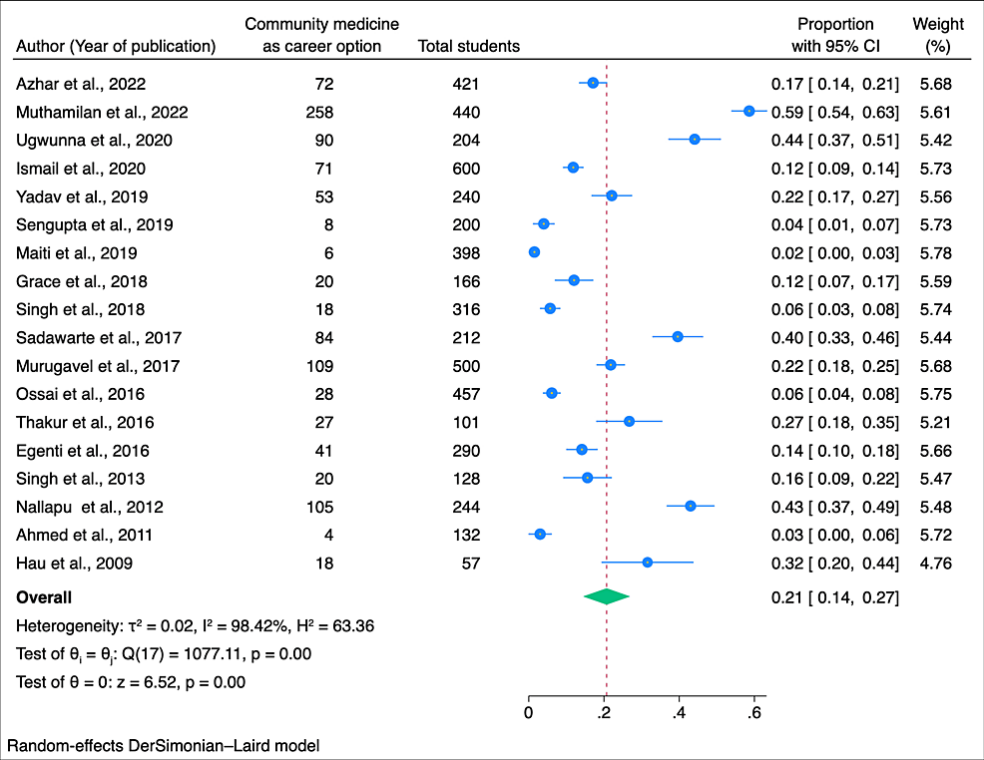
Forest plot of the meta-analysis for the willingness to take community medicine as a career option among medical students Sources: References [9-22,24-27].

A meta-analysis was conducted to assess the willingness of students to pursue community medicine as a career option, utilizing data from 5106 students across the included studies. Among this cohort, 1032 students expressed a willingness to choose community medicine as their career. The pooled estimate, derived through a random-effects model, was 0.21, with a 95% CI: of 0.14 to 0.27 (Figure 2).

**Figure 3:**
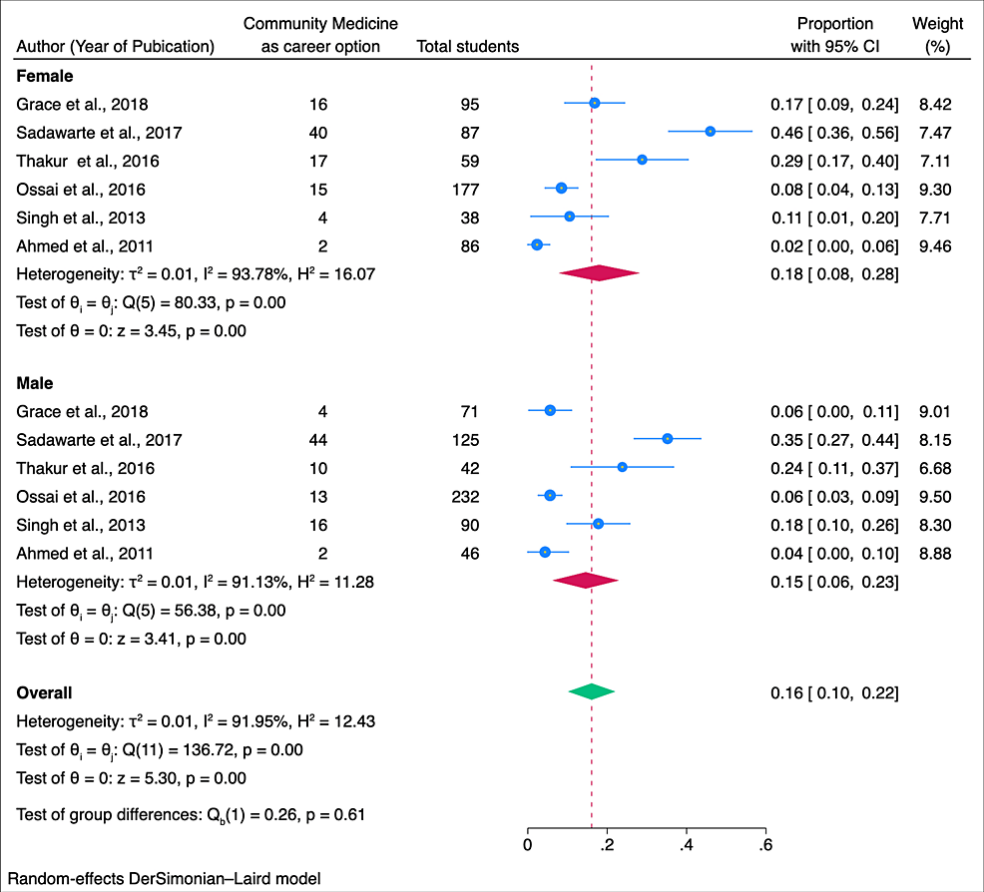
Forest plot of the meta-analysis for the willingness to take community medicine as a career option among medical students: sub-group analysis among male and female students Sources: References [17,19,21-22,26-27].

**Figure 4:**
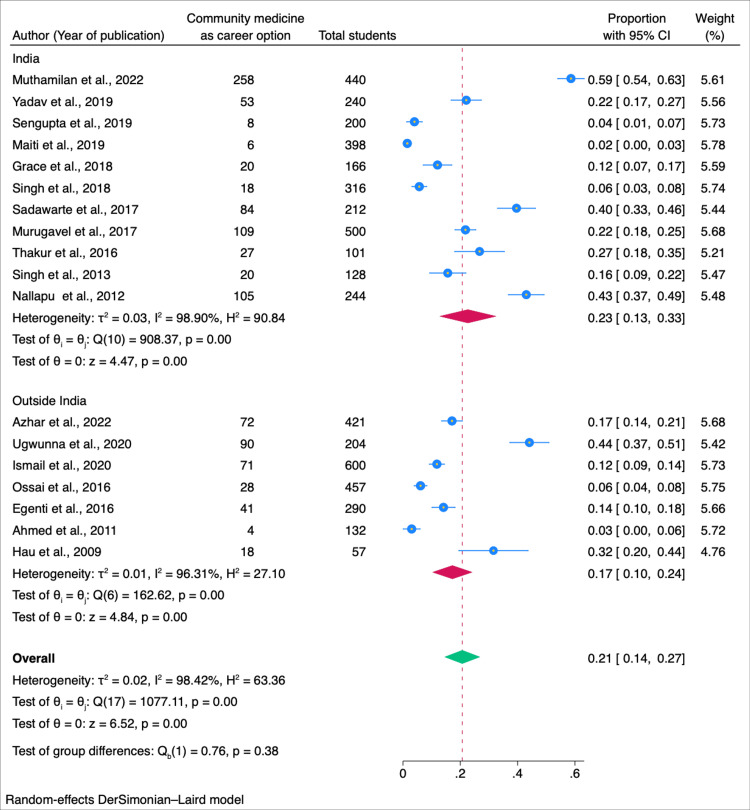
Forest plot of the meta-analysis for the willingness to take community medicine as a career option among medical students: sub-group analysis in India and abroad Sources: References [9-22,24-27].

**Figure 5:**
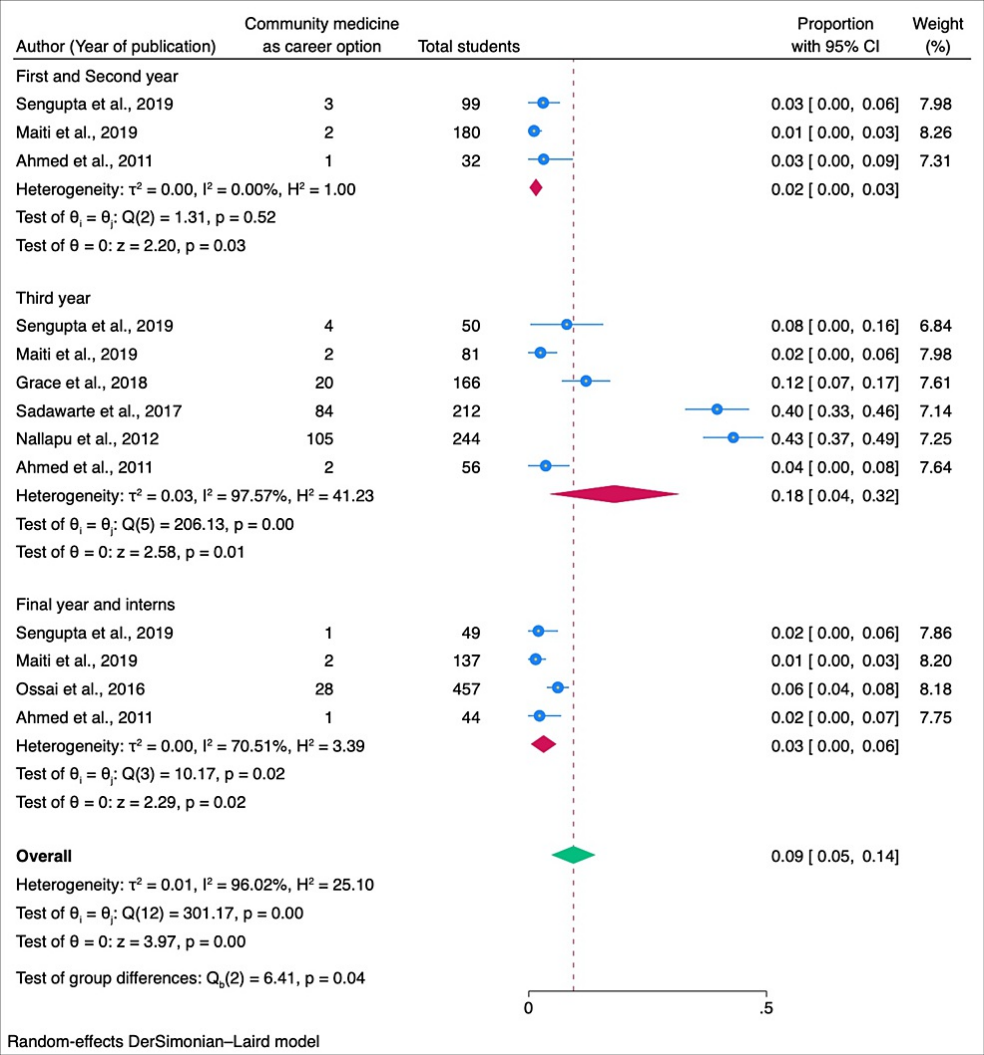
Forest plot of the meta-analysis for the willingness to take community medicine as a career option among medical students: sub-group analysis of different professional years of MBBS MBBS: Bachelor of Medicine and Bachelor of Surgery. Sources: References [7,14,15,19,24,26,27].

**Figure 6:**
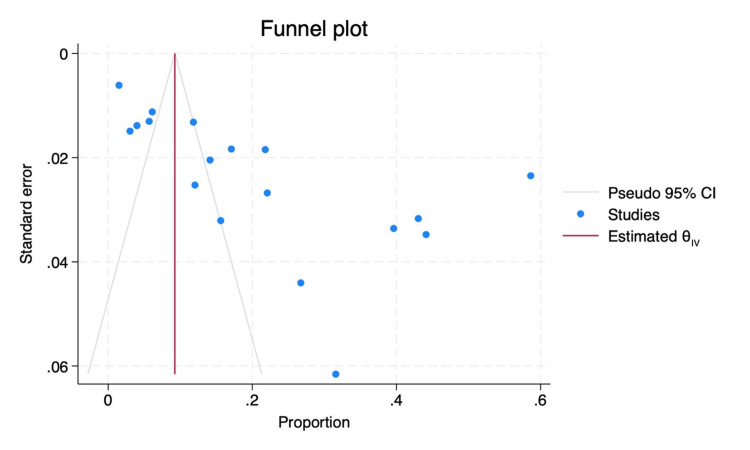
Funnel plot for publication bias

In our subgroup analysis, we explored factors influencing students' willingness to pursue community medicine as a career. Among male students, the willingness was estimated at 0.15 (95% CI: 0.06-0.23), while female students exhibited a higher proportion of 0.18 (95% CI: 0.08-0.28) (Figure 3). Geographically, studies conducted in India revealed a willingness of 0.23 (95% CI: 0.13-0.33), whereas studies conducted outside India reported a slightly lower proportion of 0.17 (0.14-0.24) (Figure 4). When considering the year of study, a combined willingness of 0.02 (95% CI: 0.00-0.03) was observed among first and second-year students, contrasting with a higher proportion of 0.18 (95% CI: 0.04-0.32) among third-year students. Fourth-year students and interns demonstrated a willingness of 0.03 (95% CI: 0.00-0.06) (Figure 5). Begg and Mazumdar's test shows no publication bias in the meta-analysis (p < 0.01) (Figure 6).

Discussion

To the best of our knowledge, this study is the first attempt to summarize the existing literature regarding the preference of medical students for opting for the subject of community medicine as a choice for a career. Globally, about 21% (14-27) of medical students considered community medicine as an option for their future persuasion. Compared to abroad (17%, 10-24), in India, this proportion is higher (23%, 13-33). The identified studies consistently highlight a prevailing inclination among medical students toward other specialty fields rather than community medicine as a future career option. The collective evidence suggests a global challenge in cultivating an interest in community medicine, prompting the need for targeted interventions and educational reforms. It is also observed that a larger proportion of third-year MBBS professional students are willing to pursue community medicine (18%, 4-32), compared to much lesser proportions in first, second, and final professional years. This is probably due to increased involvement in the subject and more exposure to teaching in the third professional year of graduation.

Several factors contributed to the unfavorable views and diminished interest in pursuing community medicine as a career. These included the perceived absence of clinical engagements, concerns about financial rewards, limited prospects for recognition and fame, comparative lack of intrigue compared to other medical subjects, frustration among postgraduate residents and stress associated with the fieldwork component, inadequate guidance from mentors (residents and faculty), challenges in comprehending the subject matter, insufficient exposure to practical work, a perceived lack of intellectual challenge and satisfaction, lower esteem from other specialty physicians, and concerns about limited job opportunities in the field. The amalgamation of these reasons highlighted the set of influences on medical students' perceptions and lack of career inclinations regarding community medicine [9,11,14,15,19,21,22,25].

Few studies also directed toward the fact that students are unclear about learning from the discipline. A high proportion of first-year MBBS students (37.8%) perceived that this subject would teach about AYUSH (Ayurveda, Yoga and Naturopathy, Unani, Siddha, and Homeopathy) medication, whereas, 16% of students believed that this subject would not teach them anything new, apart from other subjects. One-third of the students were unaware of the subject before joining MBBS [7]. Another study revealed that 17.8% of students believed that community medicine trained them to be "community workers" and 3.4% believed that it taught nothing new. Only 41.7% of third-year students reported the subject to be interesting. Almost half of the participants gave a "neutral" response when asked about their interest in learning the principles of community medicine [18]. While exploring students’ perceptions of the role of public health physicians, one of the responses was “Earlier there was no importance to PSM but from the recent COVID pandemic we learn scenario is changing because of preventive & social medicine has increased from recent COVID-19 pandemic” [6]. About 10% of medical students did not feel that prevention is an important task of practicing doctors [23].

Strength

We included studies from multiple databases. Sub-group analyses were done to explore differences among sex, MBBS professional years, India, and other countries. Publication bias was assessed.

Limitations

Our study is not beyond limitations. Researchers in several of the analyzed studies were affiliated with departments of community medicine at various medical colleges. This might have introduced information bias, as students could be inclined to provide responses deemed more favorable or aligned with the department's objectives. Our effort to systematically search for relevant literature online was exhaustive. Nevertheless, the study's scope constraints prevented the inclusion of grey literature.

Way Forward

Community medicine requires a re-branding as a subject in the medical curriculum, detaching it from the stereotypical notions. The post-pandemic world provides a perfect backdrop for this refurbishment since preventive and social medicine played vital roles during the pandemic. Firstly, there is a strong need for curriculum enhancement, integrating innovative teaching methods, and case studies that showcase the practical relevance of community medicine in health care. The scope of the subject should be told to the students by successful researchers, leaders, program managers, and academicians of the discipline. Preventive medicine is a more rewarding and cost-effective method of intervention that needs to be practiced. Collaborative efforts between medical schools and local health organizations can facilitate hands-on experiences, fostering a deeper understanding of the subject. Additionally, promoting awareness about the societal impact of community medicine through workshops and outreach programs can instill a sense of purpose and relevance among students. Mentorship programs connecting students with passionate faculty members in and across the field can inspire interest and provide guidance. Institutional support for compulsory and elective research initiatives in community medicine can also contribute to elevating its status. During the internship, more subject-centric activities like national health program evaluation and epidemiological investigations can infuse interest among young graduates. Lastly, leveraging digital platforms to disseminate success stories and the positive outcomes of community medicine practices can create a positive narrative, dispelling misconceptions and enhancing its appeal among medical students. Through these concerted efforts, we can pave the way for appreciation of the vital role of community medicine in shaping holistic healthcare professionals.

## Conclusions

The findings of this systematic review underscore the complexity of medical students' perceptions and attitudes toward community medicine. The identified challenges demand a multifaceted approach, including curriculum reforms, targeted awareness creation, and specialized career guidance programs. As the healthcare panorama continues to evolve in India and abroad, addressing these perceptions of students becomes paramount to ensure a pipeline of healthcare professionals committed to community-centric services and preventive healthcare practices. By actively engaging in the solution of these challenges, medical educators and policymakers can contribute to the vitalization of community medicine as a coveted and attractive specialty.
